# Addressing the liver progenitor cell response and hepatic oxidative stress in experimental non-alcoholic fatty liver disease/non-alcoholic steatohepatitis using amniotic epithelial cells

**DOI:** 10.1186/s13287-021-02476-6

**Published:** 2021-07-28

**Authors:** Mihiri Goonetilleke, Nathan Kuk, Jeanne Correia, Alex Hodge, Gregory Moore, Michael P. Gantier, George Yeoh, William Sievert, Rebecca Lim

**Affiliations:** 1 Centre for Inflammatory Disease, School of Clinical Sciences, Monash University, Melbourne, Victoria Australia; 2 The Ritchie Centre, Hudson Institute of Medical Research, Melbourne, Victoria Australia; 3 Gastroenterology and Hepatology Unit, Monash Health, Melbourne, Victoria Australia; 4 Department of Molecular and Translational Science, Monash University, Clayton, Victoria Australia; 5 Centre for Innate Immunity and Infectious Disease, Hudson Institute of Medical Research, Melbourne, Victoria Australia; 6 Centre for Medical Research, Harry Perkins Institute of Medical Research, QEII Medical Centre, Nedlands, Western Australia Australia; 7 School of Molecular Sciences, The University of Western Australia, Crawley, Western Australia Australia; 8 Centre for Cell Therapy and Regenerative Medicine, School of Biomedical Sciences, The University of Western Australia, Crawley, Western Australia Australia; 9 Department of Obstetrics and Gynaecology, Monash University, Melbourne, Victoria Australia

**Keywords:** Fatty liver disease, NASH/NAFLD, Liver progenitor cells, Hepatic oxidative stress, Amnion epithelial cells, Regenerative medicine

## Abstract

**Background:**

Non-alcoholic fatty liver disease is the most common liver disease globally and in its inflammatory form, non-alcoholic steatohepatitis (NASH), can progress to cirrhosis and hepatocellular carcinoma (HCC). Currently, patient education and lifestyle changes are the major tools to prevent the continued progression of NASH. Emerging therapies in NASH target known pathological processes involved in the progression of the disease including inflammation, fibrosis, oxidative stress and hepatocyte apoptosis. Human amniotic epithelial cells (hAECs) were previously shown to be beneficial in experimental models of chronic liver injury, reducing hepatic inflammation and fibrosis. Previous studies have shown that liver progenitor cells (LPCs) response plays a significant role in the development of fibrosis and HCC in mouse models of fatty liver disease. In this study, we examined the effect hAECs have on the LPC response and hepatic oxidative stress in an experimental model of NASH.

**Methods:**

Experimental NASH was induced in C57BL/6 J male mice using a high-fat, high fructose diet for 42 weeks. Mice received either a single intraperitoneal injection of 2 × 10^6^ hAECs at week 34 or an additional hAEC dose at week 38. Changes to the LPC response and oxidative stress regulators were measured.

**Results:**

hAEC administration significantly reduced the expansion of LPCs and their mitogens, IL-6, IFNγ and TWEAK. hAEC administration also reduced neutrophil infiltration and myeloperoxidase production with a concurrent increase in heme oxygenase-1 production. These observations were accompanied by a significant increase in total levels of anti-fibrotic IFNβ in mice treated with a single dose of hAECs, which appeared to be independent of c-GAS-STING activation.

**Conclusions:**

Expansion of liver progenitor cells, hepatic inflammation and oxidative stress associated with experimental NASH were attenuated by hAEC administration. Given that repeated doses did not significantly increase efficacy, future studies assessing the impact of dose escalation and/or timing of dose may provide insights into clinical translation.

**Supplementary Information:**

The online version contains supplementary material available at 10.1186/s13287-021-02476-6.

## Background

Non-alcoholic fatty liver disease (NAFLD) is the most common liver disease globally [1]. While global prevalence of NAFLD varies widely, the incidence rates in some countries are as high as 45% [2]. As many as one third of those affected by NAFLD may progress to inflammation-associated fibrotic disease and cirrhosis (non-alcoholic steatohepatitis; NASH) [3] and eventually hepatocellular carcinoma (HCC). The global prevalence of NAFLD is currently estimated at 25% [4], and in 2020, NASH replaced hepatitis C as the leading reason for liver transplantation. While patient education and lifestyle changes are considered the major tools to prevent progression of liver disease, there is currently no cure for NAFLD/NASH. With the failure of several drugs in late stage clinical development and for patients who are not transplantable, alternative options must be explored.

Emerging therapies in NASH target known pathological processes and pathways involved in the progression of the disease including inflammation, fibrosis, oxidative stress and apoptosis. Given previous reports suggesting that liver progenitor cells (LPCs) are cellular targets for malignant transformation in hepatocellular carcinoma (HCC), and the increased risk of HCC in NALFD/NASH [5–9], LPCs have been identified as a possible target for treatment [10]). Furthermore, anti-oxidants, including N-Acetylcysteine (NAC) and S-adenosylmethionine (SAMe), have been explored as potential treatment for NASH [11–13] since oxidative stress has been implicated in the progression of the LPC response [14] and pathogenesis of NASH. Recent pre-clinical studies have also explored interferon β (IFNβ) as a potential anti-fibrotic for NASH, with its ability to downregulate fibrogenic genes associated with *TGFβ-1* and *MyD88* pathways [15]. While these emerging treatments have shown varying degrees of success, there remains an urgent need to develop efficacious therapies that address the complex pathophysiologic processes implicated in NASH.

Cell-based therapies have shown promising results in the treatment and prevention of experimental NAFLD/NASH [16, 17]. Human amnion epithelial cells (hAEC) that line the amniotic sac of the placenta are non-tumorigenic and immunologically privileged. The therapeutic potential of hAECs has been explored in the setting of liver [18], lung [19], cardiac [20], epidermal [21] and neurological injury [22]. We [16, 18, 23–26] and others [27, 28] postulate that hAECs may be a promising alternative to address NAFLD/NASH either through the restriction of hepatocyte death and hepatic stellate cell activation [24] and/or through the modification of dominant macrophage phenotyp e[18, 24]. Both hAECs and their secretome have been shown to reduce chronic liver injury [23, 24]. Kuk et al. previously reported a reduction in hepatic inflammation and fibrosis in this model of experimental NASH when treated with hAECs [24]. While the anti-inflammatory and anti-fibrotic effects of hAECs have been extensively explored in carbon tetrachloride models of liver injury [18, 23–25], their effect on liver regeneration in the context of NAFLD/NASH, is poorly understood.

In this study, we investigate the influence hAECs have on the LPC response and hepatic oxidative stress in a murine model of human NASH. Previous reports show that hAECs dampen hepatic inflammation and fibrosis in experimental NASH [24], but the mechanisms through which hAECs and other cell-based modalities influence the LPC response and oxidative stress response remain unknown.

## Methods

### Ethics statement

This study was approved by Monash University Animal Ethics Committee (AE# B13/01) and conducted in accordance with the Australian Code of Practice for the Care and Use of Animals for Scientific Purposes (2006). All mice were monitored daily. The Monash Health Human Research Ethics Committee approved the collection and use of human amnion (Monash Health HREC approval numbers: 01067B, 12223B). Informed written consent was obtained from each patient prior to surgery.

### Isolation of hAECs

The hAECs were isolated as previously described [29]. Briefly, amniotic membranes were separated from underlying chorions, washed in Hanks Balanced Salt Solution (HBSS) and digested in 0.05% trypsin-EDTA (Thermo Fisher Scientific, Waltham, MA) for 1 h at 37 °C. Only batches with > 90% cellular viability were cryopreserved in liquid nitrogen for use in this study. The purity of hAEC isolates were determined by flow cytometry with EpCAM (Becton Dickinson Biosciences, Bedford, MA), CD90 (BioLegend, San Diego, CA), CD45 (Invitrogen, Carlsbad, CA) and CD31 (Becton Dickinson Biosciences, Bedford, MA) used to assess cell purity. Only batches with > 90% EpCAM (Supplemental Figure 1a) and < 1% CD90, CD45 and CD31 (Supplemental Figure 1b, c and d) positive cells were used for the following experiments. hAECs isolated from 4 placental donors were used for this animal study.

### Animals and experimental schedule

**Fig. 1:**
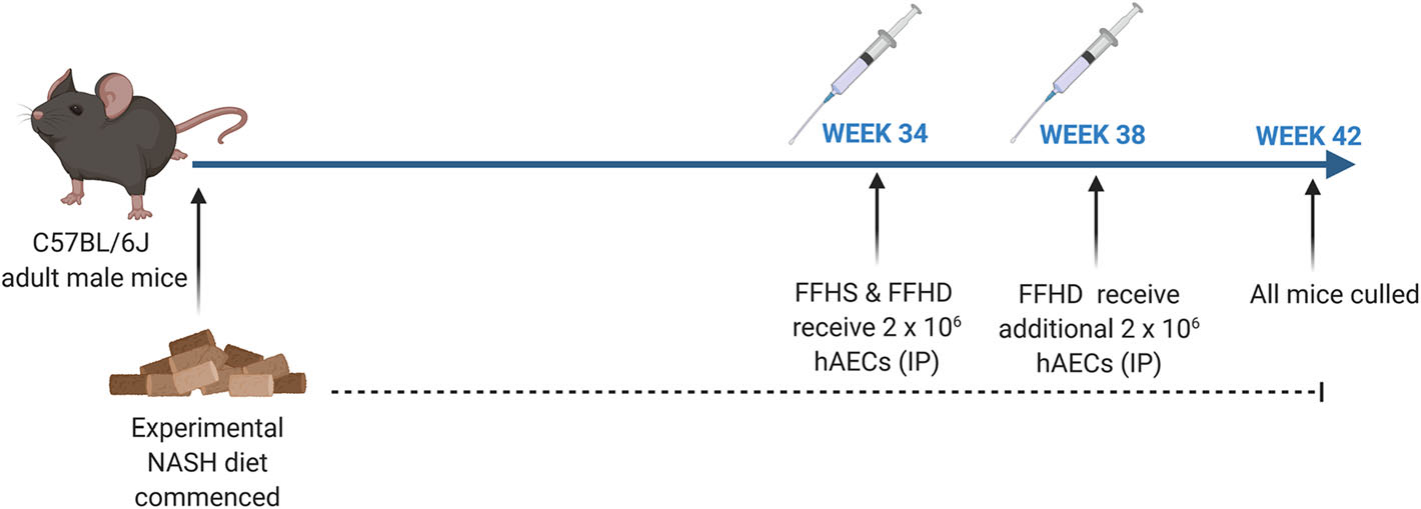
Schedule for experimental NASH diet and hAEC administration. Experimental NASH was induced in C57BL/6 J male mice using a high-fat, high fructose diet for 42 weeks. Mice received either a single intraperitoneal injection of 2 × 10^6^ hAECs at week 34 or an additional hAEC dose at week 38

Seven-week-old male C57BL/6 J mice were purchased from Monash Animal Services (Monash University, Melbourne, Australia). Mice were divided into four groups (*n* = 6–8), one group receiving ad libitum standard chow with normal water (normal), and the other three groups receiving a modified experimental NASH diet with high fructose corn syrup for 42 weeks (Table 1). The three groups receiving the experimental NASH diet were either on the diet alone (FF), received a single intraperitoneal injection of 2 × 10^6^ hAECs (FFHS) at week 34 or received two doses of 2 × 10^6^ hAECs (FFHD) at week 34 and 38 (Fig. 1). Control mice included standard chow fed and mice on the experimental NASH diet alone (FF). All mice were culled at week 42.

**Table 1:** Nutritional composition of the experimental NASH diet and standard chow

Dietary composition	Standard chow	Fast food diet
**Total fat (% weight)**	4.8%	21%
**Saturated**	0.93%	14%
**Mono-unsaturated**	0.99%	6.23%
**Polyunsaturated**	2.20%	0.77%
**Cholesterol (% weight)**	0%	2%
**Carbohydrates (% weight)**	59.4%	49.9%
**Protein (% weight)**	20%	21%
**Fibre (% weight)**	4.7%	4.7%
**Drinking water**	Tap water	High fructose water 42 g/L) 55% fructose, 45% sucrose

### Immunohistochemistry and immunofluorescence

Paraffin-embedded liver sections from all treatment groups were dewaxed, rehydrated and incubated in 10 mM sodium citrate pH 6 or 10 mM Tris-EDTA pH 9 for heat mediated antigen retrieval (Table 2). Endogenous peroxidase activity was blocked using 3% H_2_O_2_. Tissue sections were blocked with a universal protein blocking solution for 1 h and then incubated with primary antibodies overnight at 4 °C as per Table 2. Tissue sections were then washed three times and incubated with secondary antibodies (Table 2) for 1 h. For PanCK, antibody binding was detected using Vectastain ABC HRP kit (Vector Laboratories, Meadowbrook, QLD, Australia) followed by DAB chromogen (Dako, Mulgrave, Victoria, Australia). Sections for MPO, CD45, NIMP-R14 and HO-1 were incubated with DAPI (Sigma–Aldrich, St. Louis, MO, USA) for 10 min. Data are presented as number of cells per field of view (PanCK) or percentage (%) positive cells per five non-overlapping fields at × 20 magnification (CD45, NIMP-R14, MPO and HO-1) normalised to the number of DAPI positive cells using Image J (v1.53c, National Institutes of Health, USA).

**Table 2:** Immunohistochemistry and immunofluorescence antigen retrieval, primary and secondary antibodies

Antigen retrieval	Primary antibody	Secondary antibody
10 mM sodium citrate (pH 6)	**Wide spectrum screening cytokeratin (Pan-CK)** (Z0622, Dako, Mulgrave, Victoria, Australia, 1:200)	Biotinylated goat anti-rabbit IgG (BA-100, Vector Laboratories, Meadowbrook, QLD, Australia, 1:150)
	**Rabbit myeloperoxidase (MPO)** (ab45977, Abcam, Cambridge, MA, USA, 1:100)	Donkey anti-rabbit 568 (Alexa Fluor conjugates, Life Technologies, Frederick, MD, USA, 1:500)
	**Rabbit CD45** (ab10558, Abcam, Cambridge, MA, USA, 1:100)	Goat anti-rabbit 647 (Alexa Fluor conjugates, Life Technologies, Frederick, MD, USA, 1:500)
Tris-EDTA buffer (pH 9)	**Rat anti-neutrophil (NIMP-R14)** (ab2557, Abcam, Cambridge, MA, USA, 1:100)	Goat anti-rat 488 (Alexa Fluor conjugates, Life Technologies, Frederick, MD, USA, 1:100)
	**Rabbit recombinant anti-heme oxygenase 1 (HO-1)** (ab52947, Abcam, Cambridge, MA, USA, 1:100)	Donkey anti-rabbit 568 (Alexa Fluor conjugates, Life Technologies, Frederick, MD, USA, 1:500)

### RNA isolation and RT-PCR

Total RNA was isolated from mouse liver tissue or cultured cells using the RNeasy mini-kit according to the manufacturer’s instructions (Qiagen Pty Ltd, Hilden, Germany). cDNA was synthesised using the High-Capacity Reverse Transcription Kit (Applied Biosystems, CA, USA) and amplified using *Power* SYBR™ Green PCR Master Mix (Applied Biosystems, CA, USA) for qPCR. Quantitative RT-PCR (QuantStudio Real-time PCR system) was used to examine gene expression levels of *Nox2*, *Nox4*, *Sting*, *Il-6*, *Tweak*, *Ifnγ*, *Ifnβ*, *Rsad2*, *Ifit1*, *Ifih1* and *Isg15* (Table 3). Data were normalised to the housekeeping gene *18 s* with fold change calculated using delta cycle-threshold method [30]. Melting curves were used in each run to confirm specificity of amplification.

**Table 3:** Real-time quantitative PCR primers

	PCR primers	Sequence
**MOUSE**	*mNox2*-FWD	TGT CAT TCT GGT GTG GTT GG
	*mNox2*-REV	GCA GCA GGA TCA GCA TAC AG
	*mNox4*-FWD	CCA GAA TGA GGA TCC CAG AA
	*mNox4*-REV	ACC ACC TGA AAC ATG CAA CA
	*mSting*-FWD	CTA CAT TGG GTA CTT GCG GTT
	*mSting*-REV	GCA CCA CTG AGC ATG TTG TTA TG
	*mIl-6*-FWD	ATG GAT GCT ACC AAA CTG GAT
	*mIl-6*-REV	TGA AGG ACT CTG GCT TTG TCT
	*mTweak*-FWD	TTG GCC TCC TGC TGG TCG TGG TCA
	*mTweak*-REV	CTC CCG GCG GTC CTC TGC TGT CA
	*mIfnγ*-FWD	GCG TCA TTG AAT CAC ACC TG
	*mIfnγ*-REV	TGA GCT CAT TGA ATG CTT GG
	*mRn18s*-FWD	GTA ACC CGT TGA ACC CCA TT
	*mRn18s*-REV	CCA TCC AAT CGG TAG TAG CG
	*mIfit1*-RT-FWD	GAG AGT CAA GGC AGG TTT CT
	*mIfit1*-RT-REV	TCT CAC TTC CAA ATC AGG TAT GT
	*mIfnβ1*-FWD	CCC TAT GGA GAT GAC GGA GA
	*mIfnβ1*-REV	CCC AGT GCT GGA GAA ATT GT
	*mRsad2*-FWD	CTG TGC GCT GGA AGG TTT
	*mRsad2*-REV	ATT CAG GCA CCA AAC AGG AC
	*mIsg15*-FWD	CAA TGG CCT GGG ACC TAA AG
	*mIsg15*-REV	TAA GAC CGT CCT GGA GCA CT
	*mIfih1*-FWD	TCT TGG ACA CTT GCT TCG AG
	*mIfih1*-REV	TCC TTC TGC ACA ATC CTT CTC

### iMACs and BMOLs co-cultured with hAECS

Immortalised mouse macrophages (iMACs) and bipotential murine oval liver cells (BMOLs), a mouse LPC cell line, were co-cultured with hAECs from 4 donors, in 0.4 μm transwell inserts, in 6-well plates at a 1:5 ratio. Wells containing only iMACs or BMOLs served as negative controls and were maintained in DMEM:F12 supplemented with 5–10% FBS or DMEM:F12 supplemented with 30 ng/mL IGF-II, 50 ng/mL EGF, 10 μg/mL insulin, 100 U/mL penicillin and streptomycin and 5–10% FBS, respectively [26]. Cells treated with 5,6-dimethylxanthenone-4-acetic acid (DMXAA-D5817, Sigma–Aldrich, St. Louis, MO, USA) served as positive controls for cGAS-STING activation. Cells treated with inflammatory cytokines IFNγ and TNFα, 24 h before hAEC treatment, served as controls for inflammation. Cultures were maintained at 37^°^C in 95% humidity and 5% CO_2_ for 2 h prior to RNA isolation.

### Data analysis

Data were analysed using GraphPad Prism version 6.0 software (GraphPad Software, San Diego, CA, USA). Murine studies were conducted with 6–8 animals in each group. One way analysis of variance with Dunn's post-hoc test for multiple comparisons was performed. Differences were considered statistically significant when *p* < 0.05. Data are presented as mean ± standard error of mean.

## Results

### hAECs reduced LPC response in experimental non-alcoholic steatohepatitis

**Fig. 2:**
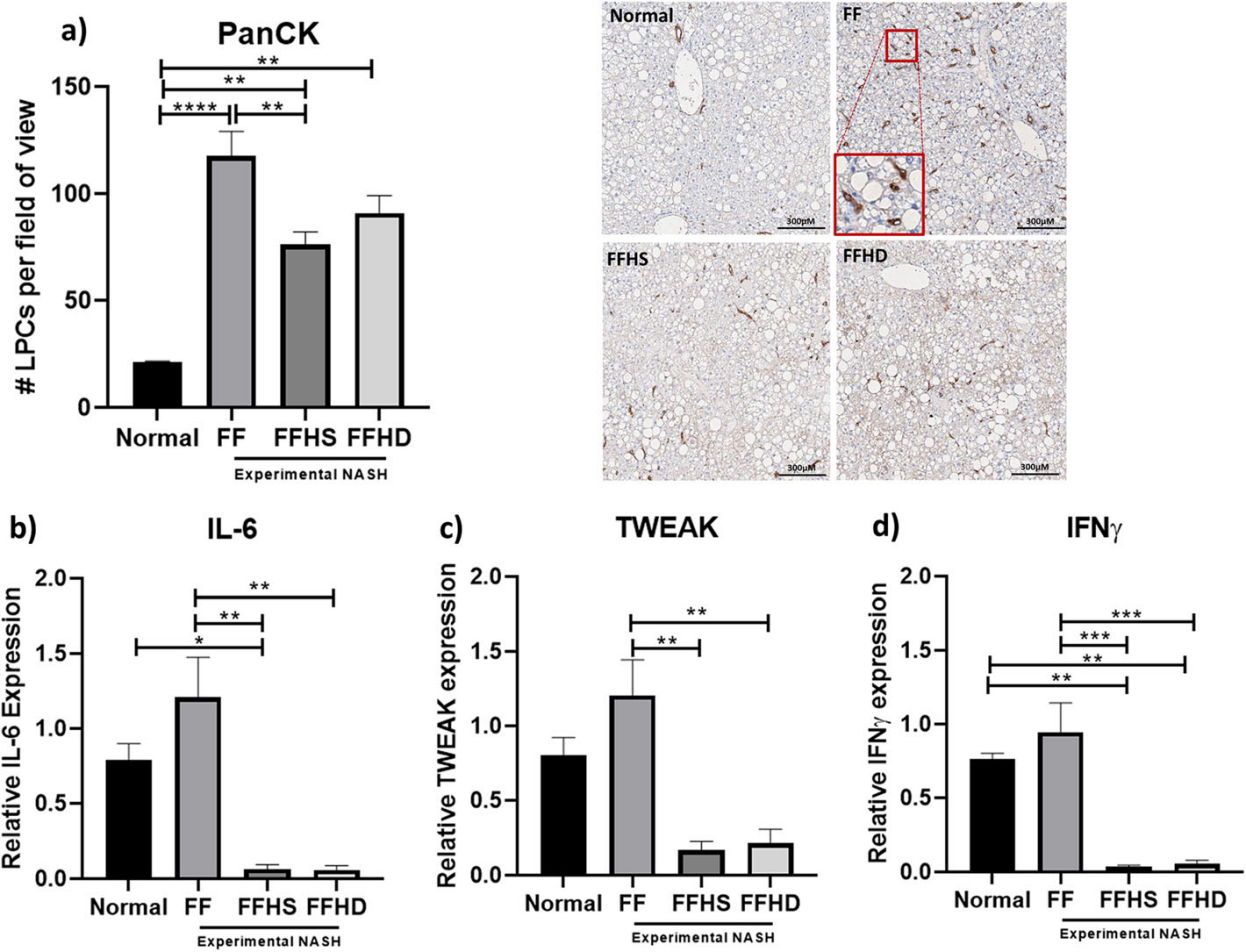
The effect of hAECs on liver progenitor cell numbers and liver progenitor cell mitogens, IL-6, TWEAK and IFNγ. All mice on the NASH diet, (FF, FFHS and FFHD) had a significantly greater number of liver progenitor cells (LPC) compared with normal mice (**a**). Within the NASH diet groups, mice treated with a single dose of hAECs (FFHS) had significantly fewer LPCs than FF mice (**a**). Compared with FF mice, FFHS and FFHD mice had significantly lower expression levels of *Il-6* (**b**), *Tweak* (**c**), and *Ifnγ* (**d**). Compared with normal mice, *Il-6* gene expression was significantly lower in FFHS mice (**b**) and *Ifnγ* gene expression was significantly lower in mice treated with both a single and double dose of hAECs (**d**). Magnification: × 10, scale bar = 300 μm; red box indicating selected area at × 20 magnification (**a**). *P < 0.05, **P < 0.01, ***P < 0.001. FF, NASH diet alone; FFHD, NASH diet with double hAEC dose; FFHS, NASH diet with single hAEC dose

The experimental NASH diet significantly increased (5.5-fold) the number of LPCs (PanCK +cells) in the liver (Normal vs. FF; 21.47 ± 0.18 vs. 117.8 ± 11.5, Fig. 2a, *p* < 0.0001). A single dose of hAECs significantly decreased the number of LPCs (FF vs. FFHS; 117.8 ± 11.5 vs. 76.27 ± 5.9, Fig. 2a, *p* = 0.005), but no greater reduction was seen in the group that received a second dose of hAECs. It is worth noting that hAEC administration did not return LPC numbers to control levels. The number of PanCK+ cells in the FFHS and FFHD groups remained elevated compared to healthy controls (normal vs. FFHS; 21.47 ± 0.18 vs. 76.27 ± 5.9, Fig. 2a, *p* = 0.0088; normal vs. FFHD; 21.47 ± 0.18 vs. 91.07 ± 8.07, Fig. 2a, *p* = 0.0019).

Next, we assessed changes to transcriptional levels of known LPC mitogens in whole mouse liver tissue. Here, we observed that compared with FF mice, a single dose of hAEC reduced transcription of *Il-6* by 20-fold (FFHS vs. FF; 0.06 ± 0.03 vs. 1.2 ± 0.27, Fig. 2b, *p* = 0.001) and by 24-fold in the FFHD group (FFHD vs. FF; 0.05 ± 0.029 vs. 1.2 ± 0.27, Fig. 2b, *p* = 0.002). The transcription of *Tweak* was reduced by 7-fold in the FFHS group (0.17 ± 0.06 vs. 1.2 ± 0.24, Fig. 2c, *p* = 0.001) and was 5-fold lower in the FFHD group (0.22 ± 0.9 vs. 1.2 ± 0.24, Fig. 2c, *p* = 0.002) compared to the fast food only group. Furthermore, transcriptional levels of *Ifnγ* were 23-fold lower in the FFHS group (0.04 ± 0.009 vs. 0.94 ± 0.2, Fig. 2d, *p* = 0.0003) and 15-fold lower in the FFHD group (0.06 ± 0.02 vs. 0.94 ± 0.2, Fig. 2d, *p* = 0.0007) compared to the FF group.

### hAECs reduced neutrophil infiltration and hepatic oxidative stress

**Fig. 3:**
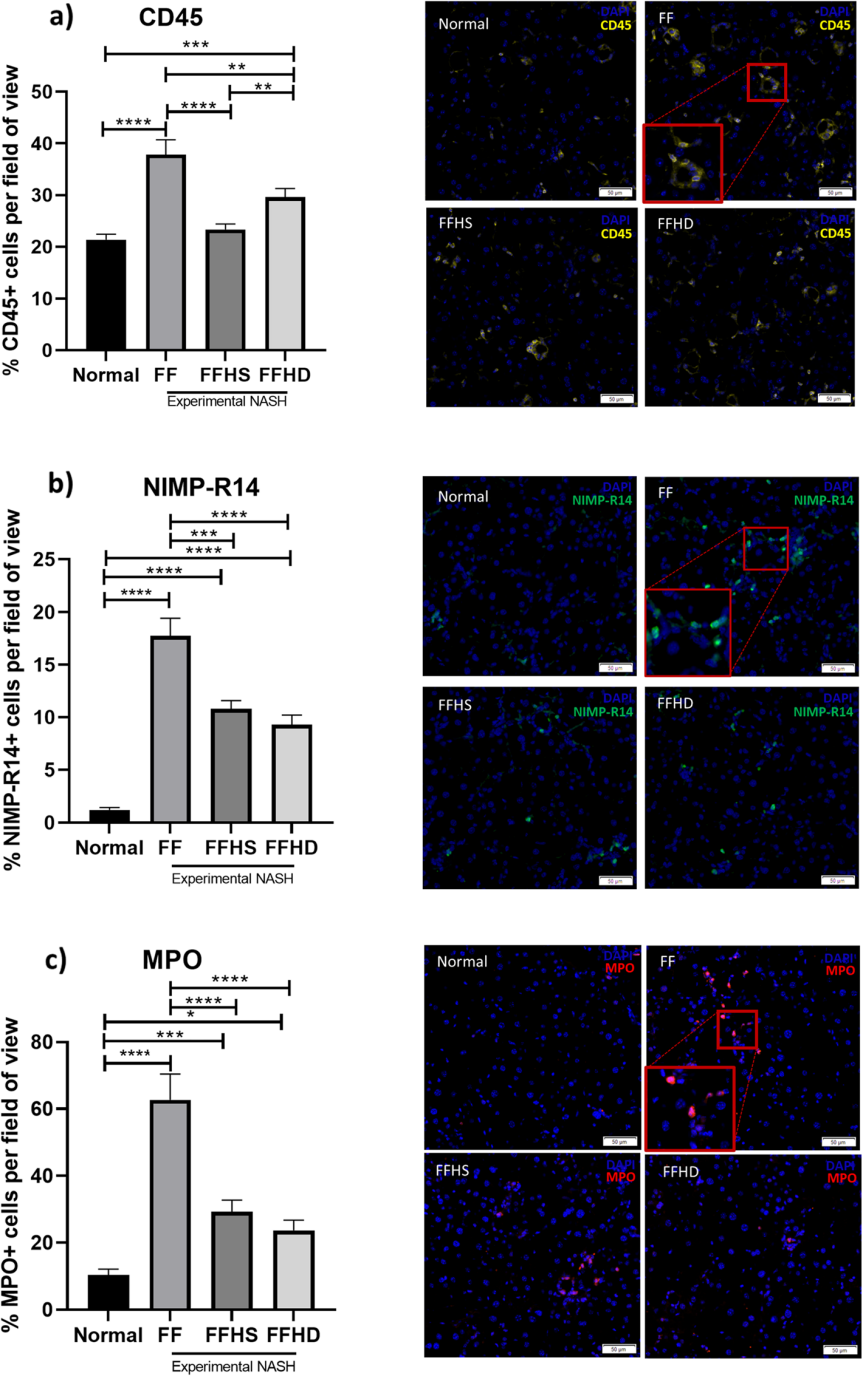
hAECs reduce levels of hepatic leukocytes, neutrophil infiltration and myeloperoxidase production. Compared with normal mice, mice on the NASH diet only (FF) and mice treated with a double dose of hAECs (FFHD) had significantly more hepatic leukocytes (CD45+ cells) (**a**). Within the NASH diet groups, both FFHS and FFHD mice had significantly lower levels of hepatic leukocytes compared to untreated FF mice (**a**). Within the mice treated with hAECs, FFHS mice had significantly fewer hepatic leukocytes compared to FFHD mice (**a**). Compared with normal mice, mice on the NASH diet, had significantly more neutrophils (NIMP-R14+ cells) (**b**). Within the NASH diet groups, FFHS and FFHD mice had significantly lower levels of neutrophils compared to untreated FF mice (**b**). Compared with normal mice, mice on the NASH diet, (FF, FFHS and FFHD) had significantly greater levels of MPO activity (MPO+ cells) (**c**). Within the NASH diet groups, FFHS and FFHD mice had significantly lower levels of MPO activity compared to untreated FF mice (**c**). Magnification: × 10, scale bar = 50 μm; red box indicating selected area at × 20 magnification. **P < 0.01, ***P < 0.001, ****P < 0.0001. MPO, myeloperoxidase

Given previous reports on the role of neutrophil infiltration [31] and the central role of oxidative stress in NASH, we next evaluated the effect of hAECs on these aspects of liver pathology. We observed a significant increase in leukocytes (CD45+ cells) in the experimental model of NASH (FF vs. normal; 37.9 ± 2.9% vs. 21.3 ± 1.08%, Fig. 3a, *p* < 0.0001). Furthermore, significantly lower levels of CD45+ cells were observed in the FFHS group (23.25 ± 1.03% vs. 21.3 ± 1.08%, Fig. 3a, *p* < 0.0001) and the FFHD group (29.61% ± 1.7 vs. 21.3 ± 1.08%, Fig. 3a, *p* = 0.0076) compared to the FF group. Notably, numbers of CD45+ cells were significantly lower in the FFHS compared to the FFHD group (23.25 ± 1.03% vs. 29.61% ± 1.7, Fig. 3a, *p* = 0.001). Further, we noted a significant increase in NIMP-R14+ neutrophils in the FF group compared to the control group (17.8 ± 1.7% vs. 1.2 ± 0.2%, Fig. 3b, *p* < 0.0001). Compared to the FF group, the number of NIMP-R14+ neutrophils was significantly reduced in both the FFHS (10.81 ± 0.8% vs. 17.8 ± 1.7%, *p* = 0.0001) and FFHD group (9.3 ± 0.9% vs. 17.8 ± 1.7%, Fig. 3b, *p* < 0.0001). This coincided with changes in hepatic MPO levels, where we observed a significant increase in the number of MPO+ cells compared to control (62.7 ± 7.7% vs. 10.3 ± 1.8%, Fig. 3c, *p* < 0.0001). Further, the number of MPO+ cells was significantly reduced in the FFHS (29.3 ± 3.5% vs. 62.7 ± 7.7%, Fig. 3c, *p* < 0.0001) and FFHD groups (23.7 ± 3.08% vs. 62.7 ± 7.7%, Fig. 3c, *p* < 0.0001).

**Fig. 4:**
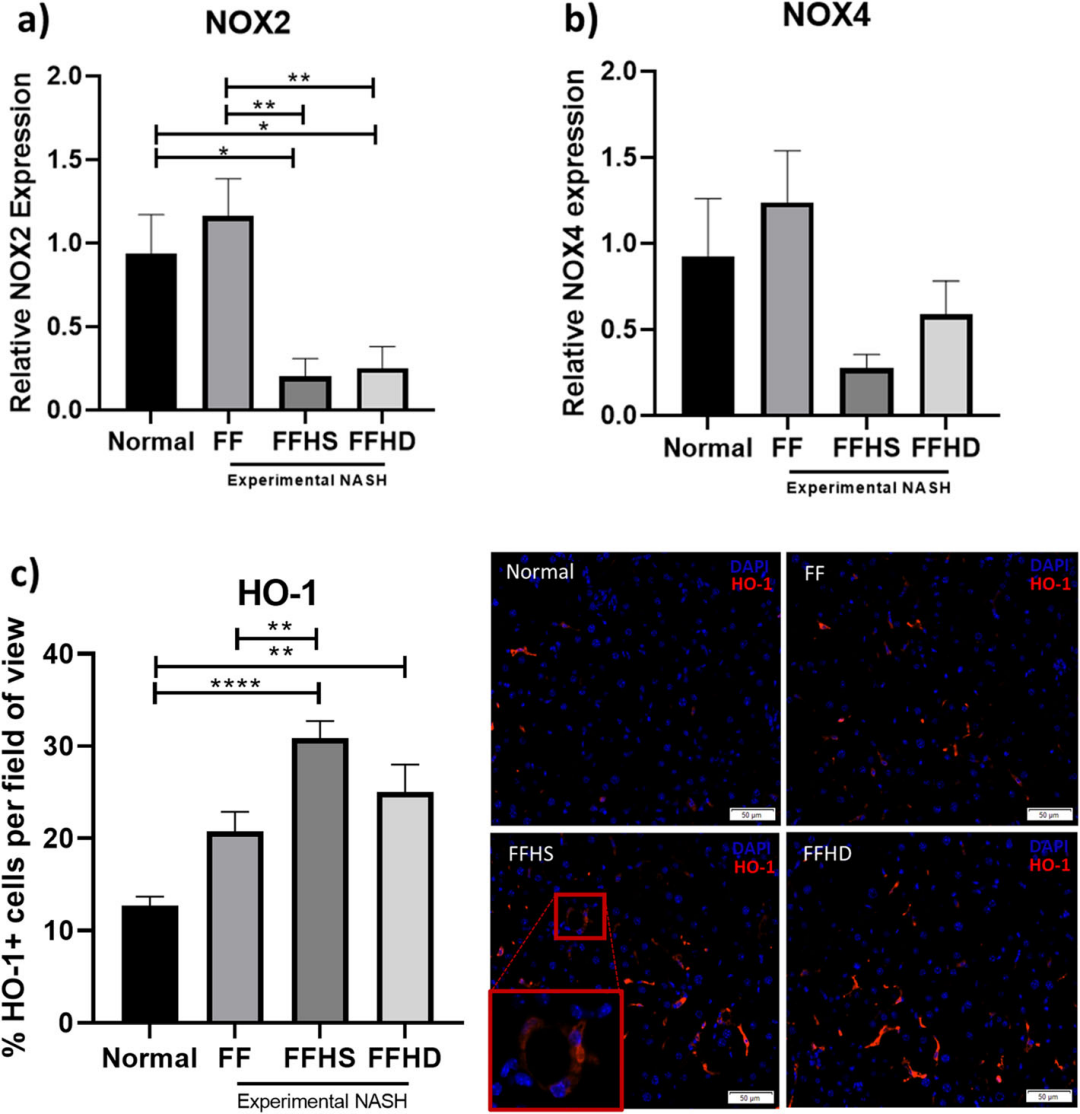
Antioxidant effects of hAECs in experimental NASH. Compared with normal mice, FFHS and FFHD mice had significantly lower expression levels of *Nox2* (**a**). Within the NASH diet groups, FFHS mice had significantly lower expression levels of *Nox2* (**a**). *Nox4* gene expression demonstrated a trend towards lower levels in hAEC exposed mice but did not reach statistical significance (*P* = 0.10) (**b**). Compared with normal mice, FFHS and FFHD mice had significantly greater levels of HO-1 activity (HO-1+ cells) (**c**). FFHS mice had significantly higher levels of HO-1 activity compared to untreated FF mice. Magnification: × 10, scale bar = 50 μm; red box indicating selected area at × 20 magnification. *P < 0.05, **P < 0.01, ****P < 0.0001. HO-1, Heme-oxygenase

We further investigated the effect of hAECs on oxidative stress by assessing the relative expression of *Nox4* and *Nox2*. We observed a 6-fold reduction in the expression levels of NOX2 in the FFHS group (0.2 ± 0.1 vs. 1.2 ± 0.22, Fig. 4a*p* = 0.003) and a 4.8-fold reduction in the FFHD group (0.25 ± 0.13 vs. 1.2 ± 0.22, Fig. 4a, *p* = 0.005). While transcriptional levels of NOX4 were lower in hAEC treated mice, this difference was not statistically significant (Fig. 4b, *p* = 0.10). We next assessed HO-1 production and found that, compared to the FF only group, there was a significant increase in expression of HO-1 in mice treated with a single dose of hAECs (20.72 ± 2.1 vs. 30.8 ± 1.9%, Fig. 4c, *p* < 0.0001) but not a double dose of hAECs. Notably, there was no significant difference in HO-1+ cells between the FFHD and FF group.

### hAECs increased total levels of hepatic IFNβ

**Fig. 5:**
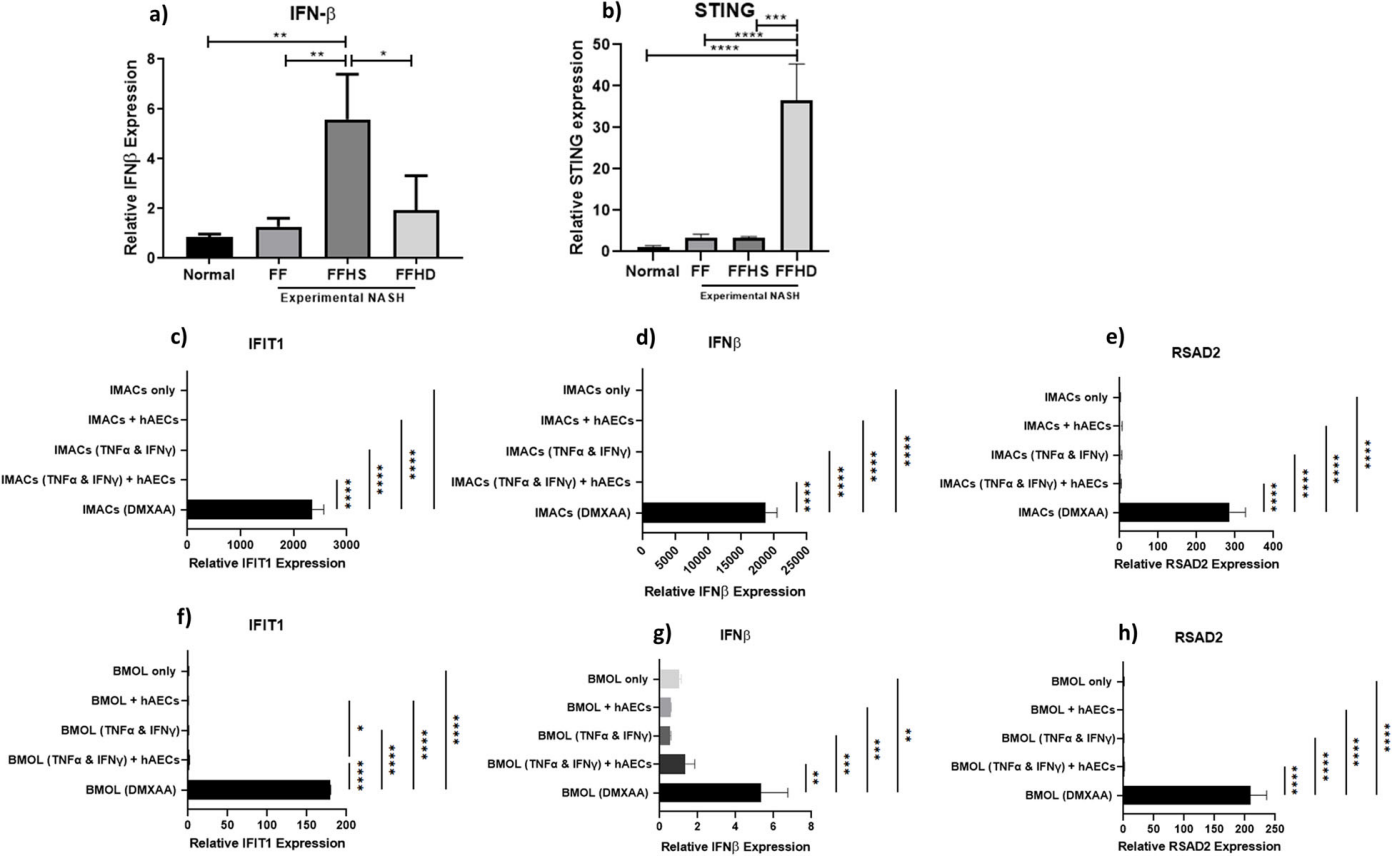
The effect of hAECs on hepatic IFN-β, STING levels and the c-GAS-STING pathway. Compared with normal mice, FFHS mice had significantly higher expression levels of IFN-β (**a**). Within the experimental NASH diet groups, only FFHS mice had significantly higher expression levels of *Ifn-β* (**a**). The *Ifn-β* expression levels were significantly higher in FFHS mice compared to FFHD mice. Compared with normal mice, FF, FFHS and FFHD mice had significantly higher expression levels of *Sting* (**b**). cGAS-STING activation was measured through *Ifit1*, *Ifn-β* and *Rsad2* gene expression in iMACs and BMOLs co-cultured with hAECs. 5,6-Dimethylxanthenone-4-acetic acid (DMXAA) treated iMACs and BMOLs served as positive controls. Compared to untreated IMACs, there was no significant difference in *Ifit1* (**c**), *Ifn-β* (**d**) and *Rsad2* (**e**) in hAEC treated iMACs. Compared to untreated BMOLs, there was no significant difference in *Ifit1* (**f**), *Ifn-β* (**g**) and *Rsad2* (**h**) in hAEC treated BMOLs. *P < 0.05, **P < 0.01, **P < 0.001, ****P < 0.0001. iMACs, immortalised macrophages; BMOLS, bipotential murine oval liver cells

Hepatic levels of IFNβ were increased by 4-fold in the FFHS group compared to the FF group (1.3 ± 0.34 vs. 5.6 ± 1.8, Fig. 5a, *p* = 0.005). Interestingly, this was not observed in the FFHD mice, with a significant difference in the hepatic levels of IFNβ between the FFHS and FFHD groups (5.5 ± 1.8 vs. 1.9 ± 1.3, Fig. 5a, *p* = 0.03). No significant differences were observed in the expression of several IFN-induced genes (*Rsad2*, *Ifit1*, *Isg15* and *Ifih1*) between treatment groups (data not shown). Furthermore, the expression of STING in the FFHD group was 10-fold higher than the FF group (36.6 ± 8.7 vs. 3.5 ± 0.8, Fig. 5b, *p* < 0.0001). In order to investigate whether the increase in hepatic IFN-β in the FFHS group was due to a direct effect on c-GAS-STING, we performed a series of in vitro studies, co-culturing hAECs with iMACs or BMOLs. Here, we observed that co-culturing with hAECs did not significantly alter the expression of IFIT1 (Fig. 5c), IFNβ (Fig. 5d) and RSAD2 (Fig. 5e) in iMACs. Similarly, co-culturing with hAECs did not significantly alter the expression of IFIT1 (Fig. 5f), IFNβ (Fig. 5g) and RSAD2 (Fig. 5h) in BMOLs. These findings suggest that the increase in hepatic IFN-β expression in the FFHS group was unlikely to be due to a direct effect of hAECs on iMACs or BMOLs—the major responders to cGAS-STING signalling in the liver.

## Discussion

The main objective of this study was to determine the impact of hAECs on the LPC population and hepatic oxidative stress, in a murine model of human NASH. Previous studies report that that hAECs dampen hepatic inflammation and fibrosis in experimental NASH [24], but the mechanisms through which hAECs, and other cell-based modalities influence the LPC response remain unknown. Previously, Kuk et al. reported that hAEC administration reduced hepatic fibrosis by decreasing activation of hepatic stellate cells and macrophages involved in inflammation and fibrogenesis in a murine model of human NASH. hAEC treatment reduced hepatic fibrosis area, which was associated with a reduction in the number of activated hepatic stellate cells and reduced hepatic macrophage numbers. While hAECs significantly reduced hepatic inflammation and fibrosis, they did not affect the metabolic components of NASH. Kuk et al. reported no significant change in the NASH Activity Score, total cholesterol levels, serum ALT or glucose tolerance tests. Kuk et al. suggested that the hAECs had no therapeutic effect on blood glucose levels due to reduced insulin sensitivity, inherent in the experimental NASH model as it is characterised by insulin resistance. They further highlighted that previous studies on NAFLD/NASH have not shown a reduction in serum cholesterol levels or bodyweight, suggesting that as the diet remained constant throughout the experimental period, a significant change in bodyweight was not expected [24]. In this study, we demonstrate that hAECs dampen the LPC response in experimental NASH through the reduction of critical LPC mitogens including TWEAK, IL-6 and IFNγ. Interestingly, we also observed that hAEC administration reduced leucocyte and specifically neutrophil infiltration and myeloperoxidase production with concurrent increase in HO-1 production. This observation was accompanied by an increase in total levels of anti-fibrotic IFNβ which appeared to be independent of c-GAS-STING activation.

Liver progenitor cells are facultative stem cells that reside in the Canals of Hering. This niche is activated during chronic liver injury when hepatocyte proliferation is insufficient to achieve homeostasis. In a fibrogenic environment where TGFβ levels are persistently elevated, the balance is tipped towards LPC expansion as hepatocytes undergo TGFβ-induced apoptosis [32, 33]. The expansion of LPCs is mediated by mitogens that significantly overlap with pro-inflammatory cytokines released by hepatic macrophages, including tumour necrosis factor (TNF), lymphotoxin β (LTβ), interferon γ (IFNγ), interleukin 6 (IL-6) and tumour necrosis factor–like weak inducer of apoptosis (TWEAK) [5, 7, 26, 34–37]. We have shown a reduction in both LPC numbers and expression levels of the LPC mitogens IL-6, IFNγ and TWEAK in hAEC treated mice most likely related to a concomitant reduction in activated hepatic macrophages. While hAECs increased LPC proliferation in in vitro co-culture studies [26], the reduction in LPC response was again most likely achieved by hAEC reduction of hepatic macrophages [24] and their secreted LPC mitogens. We have observed this consistently in previous studies using in vivo liver and lung injury models that have demonstrated that hAECs reduce macrophage recruitment and support a predominant alternatively activated (M2) phenotype [18, 19]. It should be noted that while we observed a potent anti-inflammatory effect of hAECs through the reduction in the LPC mitogens, we saw a lesser effect on the LPC numbers. This indicates that other factors including Wnt ligands recently reported to be produced by hAECs [38] may still persist to drive the LPC response in liver homeostasis and repair [39]. Furthermore, in combination with our recent report that hAECs can promote LPC proliferation and differentiation in vitro and in the absence of inflammatory cytokines [26], these findings support the notion that hAECs support liver repair and reduce inflammation. It is also noteworthy that hAECs reduce LPC mitogens in this context given that uncontrolled proliferation and growth of LPCs can lead to the development of liver cancer [40–42].

We also assessed the impact of hAEC treatment on neutrophil infiltration and activation given the relevance of neutrophils and oxidative stress in both adults with NASH and obese children at risk of developing NASH [43, 44]. Indeed, pharmaceutical approaches have been employed to address this contributor to NASH progression [31]. The degranulation of neutrophils releases myeloperoxidase (MPO), a ROS-producing enzyme that oxidises phosphatidylcholine, creating a positive feedback loop by activating more neutrophils while also acting as a ligand for scavenger receptors, thus exacerbating fibrogenesis [45]. Here, we observed that the administration of either a single or double dose of hAECs significantly reduced neutrophil infiltration as determined by NIMP-R14 (Ly-6G/-6C) staining which coincided with a reduction in hepatic MPO levels. Since these findings were limited to immunofluorescent analysis it would be beneficial to assess the changes in leukocyte numbers and neutrophil infiltration through flow cytometry in future studies. Further to this, we observed a significant reduction in the expression levels of NADPH oxidase 2 (NOX2) in mice administered with hAECs at both doses, but no significant difference in NOX4 expression levels. NOXs are a family of enzymes known to produce ROS during liver injury [46] and have been implicated in the activation of quiescent HSCs to myofibroblasts [47]. The NOX isoforms are differentially expressed by resident liver cells. Hepatic macrophages only express NOX2, while hepatocytes and hepatic stellate cells express both NOX2 and NOX4 as well as other isoforms [47]. The reduction in NOX2 expression levels following hAEC administration may be attributed to reduced macrophage recruitment previously reported [19, 24, 48]. In addition to the reduction in ROS-producing enzymes, hAEC administration also resulted in an increase in the anti-oxidant HO-1, thus suggesting that the reduction in fibrosis, reported in our previous work [24], may have been achieved through a combination of reduced oxidative stress and improved anti-oxidant capacity. Considering the role of ROS in mediating HSC activation, hepatocyte apoptosis, inhibition of hepatocyte replication and accumulation of LPCs [14, 47], these findings are extremely encouraging for the clinical translation of hAEC as a therapy for NASH. The ability of hAECs to reduce oxidative stress that enhances oncogenic mutational events in liver cells; in particular proliferating LPCs, is important as it has been shown that attenuation of the inflammatory response reduces the incidence of HCC in mouse models of fatty liver disease [49, 50].

The severity of hepatic inflammation has been identified as an independent risk factor for fibrosis progression in NASH [51]. Interferon β (IFNβ) was suggested as a potential anti-fibrotic for NASH with its ability to downregulate fibrogenic genes associated with *TGFβ-1* and *MyD88* pathways [15]. As such, we measured total gene expression of IFNβ in the livers of NASH mice and observed that a single dose of hAECs significantly increased IFNβ where a double dose of hAECs failed to achieve this. Given the implication of the c-GAS-STING pathway in the initiation of IFNβ expression and progression of liver fibrosis [52, 53], we then assessed the transcription of STING and IFN-inducible genes in total liver lysates. Here, we observed that STING expression was only significantly increased in the livers of mice exposed to a double dose of hAECs. Furthermore, we did not observe significant expression of common IFN-induced genes in vivo. Next, we performed in vitro studies using immortalised macrophages (iMACs) and a mouse LPC cell line (BMOLs) to further investigate the role of STING signalling. The purpose of this co-culture experiment was to investigate the whether the origin of the increased IFNβ and STING levels was specific to either of these cell populations. The co-culture experiment allowed us to investigate the direct effect of the hAECs on the c-GAS-STING pathway on macrophages and liver progenitor cells independent of each other. In this experiment, DMXAA acted as a positive control to activate the c-GAS-STING pathway to confirm that the cGAS-STING pathway could be activated in iMAC and BMOL cell lines. No changes in the expression of *Ifnβ*, *Ifit1* and *Rsad2* genes in iMACs or BMOLs following co-culture with hAECs were observed. hAECs did not lead to IFN induction in vitro and did not result in the initiation of c-GAS-STING signalling. Together, these data do not support a role for hAECs in cGAS-STING activation. Additionally, increased hepatic STING transcription while coincident with the double dose of hAECs does not appear to be a direct consequence of hAEC administration. It is important to recognise that the co-culture studies were limited by use of immortalised macrophages and BMOLs. Future co-culture studies using primary cells or transcriptional profiling of flow sorted cell populations may provide further insights into the cell-cell interactions. cGAS or STING knockout cell lines and/or mouse strains would be beneficial to ascertain the impact of hAECs on the cGAS-STING pathway. This is particularly important given the growing evidence that the DNA-sensing cGAS-STING pathway is critical to NASH progression [52].

As noted previously, we did not show that a second hAEC dose consistently increased efficacy. In this study, our experimental model involved the administration of two doses of hAECs through intraperitoneal injection at week 34 and 38 of the 42 week disease model of NASH. The dosage of 2 × 10^6^ hAECs was chosen based on our previous work in the CCL_4_ chronic liver injury model [26]. The timepoints for hAEC administration were based on disease progression. By week 34, fibrosis is established in this experimental NASH model and the 4-week interval between the first and second dose is based on a published model of CCL_4_ liver injury [18]. One of the reasons that the repeated hAEC dose did not significantly increase efficacy of the treatment may be due to the long-lasting effects of hAECs, where a single dose of cells may have effects that last for up to 10 weeks post administration [54]. It may not be possible to demonstrate an additive effect if an experimental outcome is maximally suppressed following a single hAEC dose (Fig. 2b–d). In addition, hAEC efficacy may be related to route of administration. Administration of hAECs through intraperitoneal injection was based on previous studies reporting reduced inflammation, fibrosis and activation of neutrophils through this administration route. Cargonini et al. showed that hAECs reduced inflammation and fibrosis of the lungs when they were administered intratracheally, intravenously or intraperitoneally [55]. Further, we had previously reported that intraperitoneally administered hAECs modify infiltration and activation of neutrophils and neutrophil-derived myeloperoxidase via pro-resolution lipid-based mediator, Lipoxin A4 [56]. Future studies focussing on the impact of the route of administration on the hAECs mechanism of action in resolving NASH would be beneficial.

## Conclusion

In summary, we provide the first evidence that hAECs reduce the LPC response in experimental NASH. This was likely achieved through a reduction in LPC mitogens as shown in this study and possibly mediated by the reduction in hepatic macrophages observed in previous work [24]. We also report for the first time that hAEC administration reduced inflammation and hepatic levels of ROS-producing enzymes concomitant with increased anti-oxidant capacity. We observed an increase in antifibrotic IFNβ following hAEC administration that appeared to be independent of cGAS-STING pathway activation. These findings are supportive of clinical translation of hAECs as a therapy for NASH. The differences seen in this study between single versus double doses of hAECs suggests that dose escalation trials should be designed to assess optimal dose rather than maximal tolerable dose. Future studies should also assess the efficacy of repeated doses compared to increased doses.

## Supplementary Information

**Additional file 1: Supplemental Figure 1.** Representation of cell purity for clinical isolation of hAECs Isolated hAECs have a cell surface profile of > 90% EpCAM (a), < 1% CD90 (b), < 1% CD45 (c), < 1% CD31 (d) positive cells. The red and blue histograms represent the hAEC population and the negative control, respectively (a). The red and blue histograms represent the hAEC population and the positive control, respectively (b-d).

## Data Availability

The datasets used and analysed during the current study are available from the corresponding author on request.

## Abbreviations

BMOL: Bipotential murine oval liver
DMXAA: 5,6-Dimethylxanthenone-4-acetic acid
FF: Fast food diet
hAEC: Human amniotic epithelial cells
HBSS: Hanks Balanced Salt Solution
HCC: Hepatocellular carcinoma
HO-1: Heme oxygenase 1
HSC: Hepatic stellate cells
IF: Immunofluorescence
IFNβ: Interferon β
IFNγ: Interferon γ
IHC: Immunohistochemistry
IL-6: Interleukin 6
iMAC: Immortalised mouse macrophages
IP: Intraperitoneal
LPC: Liver progenitor cell
LTβ: Lymphotoxin β
MPO: Myeloperoxidase
NAC: N-Acetylcysteine
NAFLD: Non-alcoholic fatty liver disease
NASH: Non-alcoholic steatohepatitis
NOX: NADPH oxidase
ROS: Reactive oxygen species
RT-PCR: Real time quantitative polymerase chain reaction
SAMe: S-adenosylmethionine
SEM: Standard error of mean
TNF: Tumour necrosis factor
TWEAK: Tumour necrosis factor–like weak inducer of apoptosis
