# Supplementary figures and images for: Addressing the liver progenitor cell response and hepatic oxidative stress in experimental non-alcoholic fatty liver disease/non-alcoholic steatohepatitis using amniotic epithelial cells
Source: Stem Cell Res Ther. 2021 Jul 28;12:429. doi: 10.1186/s13287-021-02476-6 (PMC8317377; doi:10.1186/s13287-021-02476-6)

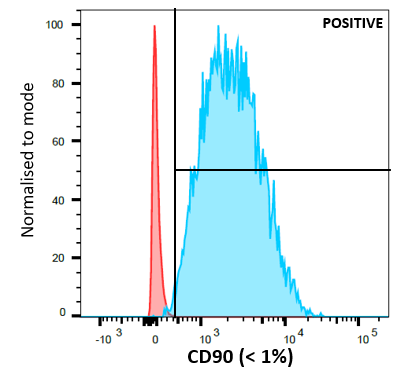

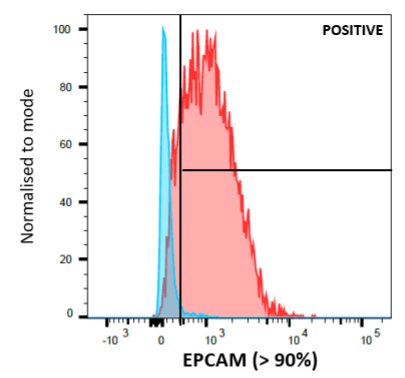


**b)**

**a)**


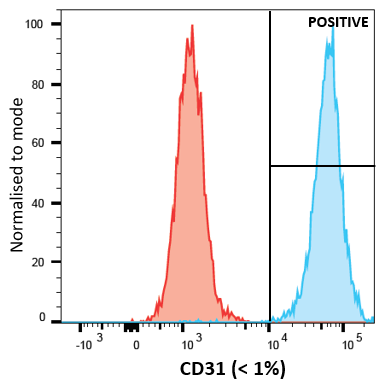

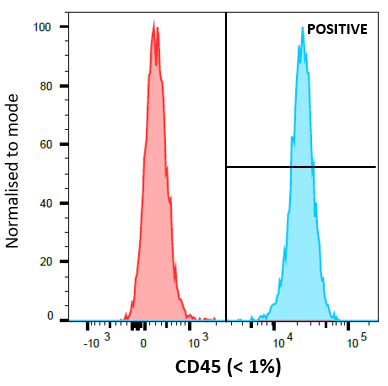


**d)**

**c)**

Supplement: Supplementary file 1 — Additional file 1: Supplemental Figure 1. Representation of cell purity for clinical isolation of hAECs Isolated hAECs have a cell surface profile of > 90% EpCAM (a), < 1% CD90 (b), < 1% CD45 (c), < 1% CD31 (d) positive cells. The red and blue histograms represent the hAEC population and the negative control, respectively (a). The red and blue histograms represent the hAEC population and the positive control, respectively (b-d). [file 13287_2021_2476_MOESM1_ESM.docx]
